# Food Addiction in the Light of DSM-5

**DOI:** 10.3390/nu6093653

**Published:** 2014-09-16

**Authors:** Adrian Meule, Ashley N. Gearhardt

**Affiliations:** 1 Institute of Psychology, University of Würzburg, Marcusstr. 9-11, Würzburg 97070, Germany; 2 Hospital for Child and Adolescent Psychiatry, LWL University Hospital of the Ruhr University Bochum, Heithofer Allee 64, Hamm 59071, Germany; 3 Department of Psychology, University of Michigan, 530 Church Street, Ann Arbor, MI 48109-1043, USA; E-Mail: agearhar@umich.edu

**Keywords:** DSM-IV, DSM-5, substance dependence, substance use disorder, gambling, food addiction, obesity, binge eating, craving, RDoC

## Abstract

The idea that specific kind of foods may have an addiction potential and that some forms of overeating may represent an addicted behavior has been discussed for decades. In recent years, the interest in *food addiction* is growing and research on this topic lead to more precise definitions and assessment methods. For example, the *Yale Food Addiction Scale* has been developed for the measurement of addiction-like eating behavior based on the diagnostic criteria for substance dependence of the fourth revision of the *Diagnostic and Statistical Manual of Mental Disorders* (DSM-IV). In 2013, diagnostic criteria for substance abuse and—dependence were merged, thereby increasing the number of symptoms for substance use disorders (SUDs) in the DSM-5. Moreover, gambling disorder is now included along SUDs as a behavioral addiction. Although a plethora of review articles exist that discuss the applicability of the DSM-IV substance dependence criteria to eating behavior, the transferability of the newly added criteria to eating is unknown. Thus, the current article discusses if and how these new criteria may be translated to overeating. Furthermore, it is examined if the new SUD criteria will impact future research on food addiction, for example, if “diagnosing” food addiction should also be adapted by considering all of the new symptoms. Given the critical response to the revisions in DSM-5, we also discuss if the recent approach of *Research Domain Criteria* can be helpful in evaluating the concept of food addiction.

## 1. Introduction

The idea that specific kind of foods may have an addiction potential and that overeating such as in binge-related eating disorders or obesity may represent a form of addicted behavior has been discussed for decades. The term *food addiction* was first introduced in the scientific literature in 1956 by Theron Randolph [1]. Although comparisons between addiction and eating behavior were sporadically drawn in the following decades [2,3,4,5,6,7,8], approaches to systematically examine and define food addiction were not pursued until the early 2000s. Particularly, a substantial increase in the number of publications using the term *food addiction* can be observed since 2009 [9].

This increased scientific interest in this topic was in part driven by the rise of neuroimaging and subsequent findings that obesity and binge eating are associated with alterations in dopaminergic signaling and food-cue elicited hyperactivation of reward-related brain areas which are comparable to processes seen in drug users [10,11]. Those findings were further complemented by animal models showing addiction-like behaviors and neuronal changes in rodents after some weeks of intermittent access to sugar [12]. In the current article, we will not go into further detail about those lines of research and refer the reader to recent works on those topics [13,14,15,16,17]. Instead, we will focus on the phenomenological similarities between substance dependence and some forms of overeating in humans.

## 2. Parallels between *Diagnostic and Statistical Manual of Mental Disorders* (DSM-IV) Substance Dependence Criteria and Overeating

The diagnostic criteria for substance dependence in the fourth revision of the *Diagnostic and Statistical Manual of Mental Disorders* (DSM-IV) included (1) tolerance, defined as consuming increasing amounts of a substance to achieve the same effects or experiencing diminished effects with continued use of the same amounts; (2) withdrawal symptoms when the substance is not consumed or using the substance to avoid withdrawal symptoms; (3) using the substance in larger amounts or over a longer period than intended; (4) a persistent desire or unsuccessful efforts to cut down substance use; (5) increased time effort to obtain or use the substance or recover from its effects; (6) reduction of social, occupational, or recreational activities because of substance use; and (7) use of the substance despite a persistent physical or psychological problem caused or exacerbated by the substance [18]. Substance dependence could be diagnosed when a clinically significant impairment or distress was present and at least three symptoms were met in the past year.

There are numerous articles in which the applicability of those DSM-IV substance dependence criteria and other features of addicted behavior to bulimia nervosa (BN), binge eating disorder (BED), obesity, or overeating in general are discussed [19,20,21,22,23,24,25,26,27,28,29,30]. However, the translation of substance dependence criteria to eating behavior is not straightforward and, as a result, there is some disagreement among researchers about the precise definitions of food addiction symptoms [31,32,33,34,35].

Although empirical evidence for the applicability of some DSM-IV addiction criteria to eating, such as tolerance and withdrawal, is mostly based on animal studies [12], all of the seven symptoms conceivably can be found in humans [26]. Compelling support for this was provided by a study by Cassin and von Ranson [36], in which almost all participants with BED received a diagnosis of substance dependence when the term *substance* was replaced with *binge eating* in a diagnostic interview. The authors noted, however, that participants’ responses may have been influenced by demand characteristics and that reliability and validity of their interview assessment was uncertain [36].

## 3. Yale Food Addiction Scale (YFAS)

In an attempt to overcome mixed definitions of food addiction symptoms and to provide a standardized measure for the assessment of food addiction, the YFAS was developed [37,38]. This 25-item instrument measures the presence of food addiction symptoms based on the DSM-IV substance dependence criteria (*i.e.*, seven symptoms). Additionally, two items assess a clinically significant impairment or distress as a result of overeating. When both a clinically significant impairment or distress is present *and* at least three out of the seven symptoms are met, then food addiction can be “diagnosed”. Prevalence rates of these food addiction diagnoses according to the YFAS range between approximately 5%–10% in non-clinical samples [37,39,40,41,42], 15%–25% in obese samples [43,44,45,46,47], and 30%–50% in morbidly obese bariatric patients or obese individuals with binge eating disorder [48,49,50,51].

The most common food addiction symptom as assessed with the YFAS is a *persistent desire or unsuccessful efforts to cut down or control eating* [42,52]. Among obese individuals, almost all participants fulfill this criterion [46,48,49,50,53]. Other commonly endorsed symptoms are *continued eating despite physical or psychological problems* and *tolerance*, particularly in obese samples (ibid.). The remaining symptoms (*consumption of large amounts or over a longer period than intended*, *spending much time obtaining food or eating or recover from its effects*, *giving up important activities*, and *withdrawal symptoms*) are less common, particularly in non-clinical samples [42,52], but are nonetheless endorsed by a substantial proportion of obese individuals [48,49,50,53].

## 4. Substance Dependence Criteria in DSM-5

In the newly revised version of the DSM, the diagnostic criteria for substance abuse and—dependence were merged such that criteria for substance use disorders (SUDs) now additionally include (1) failure to fulfill major role obligations at work, school, or home as a result of substance use; (2) continued substance use despite social or interpersonal problems caused or exacerbated by substance use; and (3) recurrent substance use in situations in which it is physically hazardous [54]. Moreover, the DSM-IV substance abuse criterion of having legal problems was dropped, but a newly created symptom of *craving*, *or a strong desire or urge to use the substance* was incorporated (Table 1). Three levels of severity can now be specified ranging from *mild* (presence of two to three symptoms) to *moderate* (presence of four to five symptoms) to *severe* (presence of six or more symptoms).

Notably, SUD symptoms also differ across substances (Table 1). For example, although there is an intoxication and withdrawal syndrome described for caffeine, the other symptoms do not apply for caffeine and, thus, there is no caffeine use disorder. *Vice versa*, although all of the eleven symptoms apply to tobacco, there is no intoxication described. Finally, there is no withdrawal syndrome described for hallucinogens, for example phencyclidine, and inhalants.

## 5. Parallels between New DSM-5 Criteria and Overeating

### 5.1. Craving

Craving refers to an intense desire to consume a substance and frequent experiences of craving are a core feature of SUDs [55]. However, the term craving does not only refer to drug-related, but also to other substances like food or non-alcoholic beverages [56]. In Western societies, individuals usually crave foods that are high in sugar or fat (or both) and, thus, highly palatable. Accordingly, the most often craved food is chocolate, followed by pizza, salty foods, ice cream and other sweets and desserts [57] (but note there also cultural differences in the types of food craved [58]). These same types of foods are more likely to be consumed in an addictive-like manner as assessed by the YFAS [39]. As such, experiences of craving are a prime example of the similarities between eating and substance use. Similarly, activation patterns of neuronal structures underlying craving experiences largely overlap across different substances, including food [15,59,60,61]. Overeating is associated with more intense and more frequent experiences of food craving. For example, higher scores on self-reported food craving measures have been found in patients with BN, BED, or obesity [62,63]. Similarly, food addiction as measured with the YFAS is also related to higher self-reported food craving [44,45,64]. Thus, the criterion of frequently experiencing craving or a strong urge to consume a substance can be translated to food and represents an important symptom in food addiction.

### 5.2. Failure to Fulfill Major Role Obligations

We are not aware of any study that specifically investigated a failure to fulfill major role obligations at work, school or home resulting from addiction-like eating. Although this may likely occur in the case of morbid obesity as a result of reduced mobility, it is questionable if this also can be a direct consequence of eating behavior. Based on the wording of the DSM-5, future studies may ask participants if they neglect things like work, school, friends, family, or household chores because of the way they eat or if they are not doing well at school or work because of the way they eat. However, we suspect that, like tobacco, this symptom may not be a core aspect of addiction-like eating due to a lack of intoxication syndrome.

**Table 1:** Substance use disorder criteria according to the *Diagnostic and Statistical Manual of Mental Disorders* (DSM-5) and possible corresponding food addiction criteria.

	Substances									Food	
	Alcohol	Caffeine	Cannabis	Hallucinogens/Phencyclidine	Inhalants	Opioids	Sedatives, hypnotics, and anxiolytics	Stimulants	Tobacco		
*Substance Use Disorder Criteria* *										*Possible Food Addiction Equivalents*	*Comment*
Substance often taken in larger amounts or over a longer period than was intended	√	×	√	√	√	√	√	√	√	Food often consumed in larger amounts or over a longer period than was intended	Empirically supported
2. Persistent desire or unsuccessful efforts to cut down or control substance use	√	×	√	√	√	√	√	√	√	2. Persistent desire of unsuccessful efforts to cut down or control food intake	Empirically supported
3. Great deal of time is spent in activities necessary to obtain or use the substance or recover from its effects	√	×	√	√	√	√	√	√	√	3. Great deal of time is spent in activities necessary to obtain or overeat on foods or recover from its effects	Plausible
**4**. **Craving, or a strong desire or urge to use the substance**	√	×	√	√	√	√	√	√	√	4. Craving, or a strong desire or urge to eat specific foods	Empirically supported
**5**. **Recurrent substance use resulting in a failure to fulfill major role obligations at work, school, or home**	√	×	√	√	√	√	√	√	√	5. Recurrent overeating resulting in a failure to fulfill major role obligations at work, school, or home	Plausible
**6**. **Continued use despite having persistent or recurrent social or interpersonal problems caused or exacerbated by the effects of the substance**	√	×	√	√	√	√	√	√	√	6. Continued overeating despite having persistent or recurrent social or interpersonal problems causes or exacerbated by the effects of specific foods	Plausible
7. Important social, occupational, or recreational activities are given up or reduced because of substance use	√	×	√	√	√	√	√	√	√	7. Important social, occupational, or recreational activities are given up or reduced because of overeating on foods	Plausible
**8**. **Recurrent substance usein situations in which it is physically hazardous**	√	×	√	√	√	√	√	√	√	8. Recurrent overeatingin situations in which it is physically hazardous	Plausible in the context of an acute health condition, but less likely to be relevant
9. Substance use is continued despite knowledge of having a persistent or recurrent physical or psychological problem that is likely to have been caused or exacerbated by the substance	√	×	√	√	√	√	√	√	√	9. Overeating is continued despite knowledge of having a persistent or recurrent physical or psychological problem that is likely to have been caused or exacerbated by overeating on foods	Empirically supported
10. Tolerance	√	×	√	√	√	√	√	√	√	10. Tolerance	Plausible
need for markedly increased amounts of the substance to achieve intoxication or desired effect										need for markedly increased amounts of food to achieve desired effect	
b. markedly diminished effect with continued use of the same amount of the substance										b. markedly diminished effect with continued use of the same amount of food	
11. Withdrawal	√	√	√	×	×	√	√	√	√	11. Withdrawal	Plausible, but hard to distinguish from energy deficit
withdrawal syndrome (differs by substance)										withdrawal syndrome when refraining from eating specific foods	
b. substance is taken to relieve or avoid withdrawal symptoms										b. specific foods are eaten to relieve or avoid withdrawal symptoms	
*Intoxication*	√	√	√	√	√	√	√	√	×	×	No intoxication

*Notes*: Criteria printed in boldface are new in DSM-5. *Empirically supported* refers to evidence that this symptom can be observed with regard to eating behavior in humans based on different assessment methods. *Plausible* refers to preliminary evidence that this symptom can be observed with regard to eating behavior based on animal models, anecdotal reports, or self-reports such as the YFAS. * A problematic pattern of substance use leading to clinically significant impairment or distress. Symptoms must occur within the past 12 months. Severity is specified as: mild (2–3 symptoms), moderate (4–5 symptoms), severe (6 or more symptoms).

### 5.3. Social or Interpersonal Problems

Social and interpersonal problems can clearly be observed in the context of eating behavior. For example, obese individuals report heightened levels of social isolation as compared to people with normal weight [65]. While this is likely a result of weight gain, it has also been found that interpersonal problems such as interpersonal distrust, social insecurity, or hostility are linked to binge eating behavior, independent of body mass [66,67]. The relationship between binge eating and interpersonal problems is likely a bidirectional one. That is, interpersonal problems may foster negative affect and earlier onset of BED, but binge eating might as well exacerbate and maintain interpersonal problems [68,69]. This is also reflected in the fact that both Cognitive-Behavioral Therapy (which focuses directly on eating behavior) and Interpersonal Psychotherapy (which focuses on interpersonal relationships) appear to be similarly effective in the treatment of BED [70,71]. Nevertheless, future studies are needed showing that addiction-like eating is causally involved in social and interpersonal problems. This may be assessed with questions like “I avoided social situations because people do not approve of the way I eat” or “I got in arguments with my family or friends because of the way I eat” in future versions of the YFAS.

### 5.4. Use in Physically Hazardous Situations 

The symptom of recurrent substance use in situations that are potentially physically hazardous mainly refers to effects of intoxication, for example, that it is dangerous to handle machines or driving a car after consumption of alcohol. Eating food, of course, does not involve intoxication. However, as described above there is also no intoxication for tobacco. Instead, it is indicated in the DSM-5 that, for tobacco, this criterion may refer to smoking in bed, which increases the risk of starting a fire. Following this line of thought, it could also be argued that this symptom could be endorsed with regard to eating when it refers to, for example, eating while driving. It is widely known that eating while driving impairs driving performance and increases the risk for crashes [72,73,74]. A further prerequisite for the applicability of this symptom to food addiction would be, of course, studies showing that patients with BN, BED, obesity, or individuals receiving a YFAS diagnosis, actually engage more often in eating while driving (or similar situations) as compared to control subjects. To our knowledge, no such studies exist yet.

Another interpretation of this symptom could be that it refers to food consumption in the context of an acute health condition associated with obesity. For example, this may refer to eating lots of sugar despite being diabetic or overeat on the wrong foods after bariatric surgery. As hazardous effects would be a result of weight gain rather than a direct consequence of eating behavior, we would argue that, like tobacco, this symptom is likely to be less relevant in food addiction because of the absence of intoxication.

## 6. Gambling Disorder and Overeating 

Besides the revised SUD criteria, gambling disorder has now been added as a non-substance-related disorder [54]. Diagnostic criteria include (1) a need to gamble with increasing amounts of money in order to achieve the desired excitement; (2) being restless or irritable when attempting to cut down or stop gambling; (3) repeated unsuccessful efforts to control, cut back, or stop gambling; (4) a preoccupation with gambling; (5) gambling when feeling distressed; (6) after losing money gambling, returning another day to get even; (7) lying to conceal the extent of involvement with gambling; (8) jeopardizing or losing significant relationships, jobs, or educational or career opportunities because of gambling; and (9) relying on others to provide money to relieve desperate financial situations caused by gambling (Table 2). Gambling disorder can be diagnosed as *mild* (four to five criteria met), *moderate* (six to seven criteria met), or *severe* (eight to nine criteria met), when symptoms were present in the past year.

Some of the gambling disorder criteria can conceivably be applied to eating behavior. For example, repeated unsuccessful efforts to control, cut back, or stop the behavior is a core feature of BN, BED, and food addiction as measured with the YFAS (see above). Moreover, studies using the YFAS consistently show that food addiction is strongly associated with a preoccupation with food and eating and with overeating when feeling distressed [37,39,48,49,64,75]. As with withdrawal syndrome in SUDs, a restlessness or irritability when attempting to cut down or stop overeating seems plausible. Using the YFAS, almost 30% of obese individuals and up to 50% of obese individuals with BED report regular experiences of such withdrawal symptoms when cutting down on certain foods [48,49,50]. However, these subjective reports are potentially biased as it may be hard for respondents to distinguish between symptoms emerging from a general energy deficit (*i.e.*, consuming not enough calories) and those that are actually associated with avoiding specific foods.

The criterion of the need to gamble with increasing amounts of money in order to achieve the desired excitement may be translated to a need to eat increasing amounts of food in order to achieve the desired satisfaction. This definition would be, thus, equal to the SUDs’ tolerance criterion, which has been shown to be endorsed by a substantial proportion (approximately 50%–60%) of obese individuals in studies using the YFAS [48,49,50]. However, this criterion may not be applicable to eating when keeping the reference to a feeling of excitement when engaging in the behavior.

Other symptoms seem transferable when substituting the term *gambling* with *overeating* (Table 2). Individuals with BN or BED usually experience marked feelings of shame and, thus, conceal their binge eating and this often involves deceiving others about the extent of involvement with overeating [76]. Jeopardizing or loss of a significant relationship, job, or educational or career opportunity may most likely occur because of weight gain. For example, there is experimental evidence showing that human resource professionals underestimate the occupational prestige of obese individuals and would less likely hire them [77]. Regarding the criterion of desperate financial situations caused by gambling, the money spent on binge foods markedly affects quality of life in individuals with BN and BED, the latter of which are in particular bothered by financial problems [78,79]. Although binge eating involves spending substantial amounts of money, actually plunging into debt or borrowing money from other people to finance overeating probably only occurs in rare cases. Finally, the symptom of returning another day to get even after losing money gambling does neither seem to be transferable to eating behavior nor to SUDs.

**Table 2:** Gambling disorder criteria according to the DSM-5 and possible corresponding food addiction criteria.

Gambling Disorder *	Possible Food Addiction Equivalents	Comment
Need to gamble with increasing amounts of money in order to achieve the desired excitement	Need to eat increasing amounts of food in order to achieve the desired satisfaction	Plausible, but not applicable when referring to excitement
2. Restlessness or irritability when attempting to cut down or stop gambling	2. Restlessness or irritability when attempting to cut down or stop overeating	Plausible, but hard to distinguish from energy deficit
3. Repeated unsuccessful efforts to control, cut back, or stop gambling	3. Repeated unsuccessful efforts to control, cut back, or stop overeating	Empirically supported
4. Preoccupation with gambling	4. Preoccupation with food and eating	Empirically supported
5. Gambling when feeling distressed	5. (Over-)eating when feeling distressed	Empirically supported
6. After losing money gambling, often return another day to get even	6. 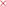	Not applicable
7. Lying to conceal the extent of involvement with gambling	7. Lying to conceal the extent of involvement with overeating	Plausible
8. Jeopardizing or loss of a significant relationship, job, or educational or career opportunity because of gambling	8. Jeopardizing or loss of a significant relationship, job, or educational or career opportunity because of overeating	Plausible
9. Relying on others to provide money to relieve desperate financial situations caused by gambling	9. Relying on others to provide money to relieve desperate financial situations caused by overeating	Plausible, but unusual

Notes: *Empirically supported* refers to evidence that this symptom can be observed with regard to eating behavior in humans based on different assessment methods. *Plausible* refers to preliminary evidence that this symptom can be observed with regard to eating behavior based on animal models, anecdotal reports, or self-reports such as the YFAS. * Persistent and recurrent problematic gambling behavior leading to clinically significant impairment or distress. Symptoms must occur within the past 12 months. Severity is specified as: mild (4–5 symptoms), moderate (6–7 symptoms), severe (8–9 symptoms).

## 7. Implications of the Research Domain Criteria for Food Addiction Research

Recently, the *Research Domain Criteria* (RDoC) have been introduced as a new approach to classifying mental illnesses, although it is important to note that the RDoC is designed as a research framework rather than an alternative diagnostic framework [80,81,82]. The RDoC approach is designed to focus on domains that reflect neurobiological, physiological, genetic and behavioral underpinnings. The current domains focus on positive valence, negative valence, cognitive functioning, social processes, and arousal/regulation [80]. Critics of the DSM suggest that the focus on “theory free” assessment has limited the incorporation of scientific advances into the diagnostic framework [82]. Thus, in its current form, the DSM may not adequately reflect knowledge gained in the areas of genetic, physiological, and neurobiological research. Although the RDoC system is not designed to be implemented as a diagnostic method in clinical settings, it is likely to be a major guiding factor in scientific evaluations of psychopathology and will hopefully improve treatment effectiveness [80].

The RDoC approach to diagnosis will also likely guide research on whether an addictive process contributes to certain types of overeating. Binge eating disorder appears to be related to many of the mechanisms implicated in addictive disorders, including elevated motivation to seek out palatable foods, greater neural activation in reward-related circuitry to high-calorie food cues, and limitations in cognitive control [23,83]. However, individuals with a BED diagnosis are not homogenous, with a subtype that is indicated by high-levels of dietary restraint and another subtype that exhibits greater negative affect, impulsivity, and overall pathology [84,85]. These two subtypes of BED could potentially be driven by different mechanisms with an addictive process possibly contributing to the latter subtype (but not the former). Thus, some (but not all individuals) with a BED diagnosis may experience an addictive response to certain foods.

Finally, one of the major proposed mechanisms underlying addiction is the ability of an addictive substance/behavior to alter underlying systems in a manner that drives problematic behavior [86]. In other words, individual risk factors (e.g., impulsivity, reward sensitivity, negative affect) interact with the addictive potential of a substance/behavior to result in pathology. As the RDoC approach highlights the importance of identifying mechanisms, examining whether certain foods or ingredients in foods are capable of altering the system in a manner that is akin to addictive substances/behaviors will be an essential line of research. There has been significant progress in this area using animal models of eating behavior [87,88,89], but research in humans is limited. Addressing this gap in the literature is extremely important for evaluating the validity of the food addiction concept. In sum, the RDoC system will be important for the evaluation of the concept of food addiction as it highlights moving beyond shared signs and symptoms and instead focuses on evaluating whether the etiology and underpinnings of addictions are contributing to compulsive food consumption.

## 8. Implications of the Revised Criteria for Food Addiction Research

### 8.1. Is Food Addiction an SUD or Behavioral Addiction?

The inclusion of gambling disorder as a behavioral addiction along with SUDs in DSM-5 necessitates a discussion if food addiction more resonates with the criteria used for SUDs or with those used for gambling disorder. The term food addiction a priori implies that consumption of a substance (or in this case, several substances that combine as food) is essential to this kind of addiction. Research into what foods (or ingredients in certain foods) may be addictive is in its nascent stages. It is possible that some symptoms of addiction may be prominent with certain kinds of food. For example, animal models suggest that sugar may be more associated with withdrawal symptoms than fat [87]. It is also possible that there may be symptoms unique to an addictive-response to highly processed foods relative to drugs of abuse, but future research is needed. Besides the potential relevance of specific types of foods/ingredients, however, research has also highlighted that specific eating patterns (or *eating topography*) may be necessary in order that food develops its addictive properties. Specifically, it has been found that food addiction symptoms particularly can be observed when high-calorie foods are consumed with alternating periods of restriction and bingeing [12,22].

Likewise, food addiction shows parallels to both SUDs and gambling disorder. We would argue, however, that the SUD criteria could more unambiguously be translated to food and eating. For example, gambling disorder includes symptoms that specifically refer to the money lost during gambling (criteria 1, 6, and 9), which hardly can be applied to eating. Thus, although food addiction may represent a mixture of an SUD and a behavioral addiction, we conclude that the DSM-5 SUD criteria rather than those for gambling disorder should guide future research on food addiction.

### 8.2. Will Using the New SUD Criteria Increase or Decrease the Prevalence of Food Addiction?

In DSM-IV, substance dependence could be diagnosed when at least three symptoms were presented. This threshold was substituted by different severity levels and SUD with mild severity can now be diagnosed when at least two symptoms are present. This will likely increase prevalence for food addiction. For instance, a recent study by Curtis and Davis [90] used a semi-structured interview among obese individuals with and without BED focusing on their experience of their binge eating or overeating, respectively. They found that all participants with BED (*n* = 12) and 42% (5 out of 12) of those without BED met the mild severity criteria for SUD, which exceeds prevalence estimations of food addiction based on the YFAS [91,92]. Notably, participants rarely mentioned three out of the four new criteria as being core problems associated with their eating [90]. In line with findings of studies using the YFAS, two of the most often reported symptoms were *taken in larger amounts of food* and *unsuccessful attempts to cut down*, regardless if individuals had BED or not. In addition, obese individuals with BED most often fulfilled the criteria of *continued use despite problems* and frequent experiences of *craving* [90].

Thus, using the mild severity threshold may overestimate food addiction prevalence, as most individuals with obesity, but also many non-obese individuals who struggle with dieting, overeating, and overweight may endorse at least two symptoms. Additionally, individuals with clinically relevant binge eating will likely receive a diagnosis with at least *moderate severity* (four to five symptoms), which is partly due to the inclusion of the new craving criterion. The DSM-5 indicates that mental disorders, such as addiction, result in clinically significant impairment or distress [54]. In addition to symptoms, the YFAS also assesses whether clinically relevant levels of difficulties are present [37]. It may be important to consider clinical severity regarding the application of DSM-5 to addictive-like eating as an exclusion criterion.

### 8.3. Is a Revision of the YFAS Necessary?

Given the large overlap between the old and new SUD criteria, we would argue that the YFAS will still be useful for future examinations of food addiction. However, a new version is likely needed to evaluate the questions raised above and, thus, is currently under development. A crucial aspect here is the importance of examining thresholds, particularly for the craving criterion. Although more frequent and intense food cravings are associated with binge eating or YFAS scores [44,45,64,90], food craving per se is a common experience in humans that is not associated with disordered eating or significant distress in most individuals [93]. Thus, simply asking participants if they sometimes experience food craving or not will likely result in high sensitivity, but low specificity for diagnosing food addiction.

## 9. Conclusions

Research on the DSM-IV diagnostic criteria for substance dependence shows that they can be translated to eating behavior and that many individuals with obesity and/or BED fulfill those criteria based on self-report measures such as the YFAS. With regard to the newly added criteria in DSM-5, one study shows that three out of four symptoms may be less relevant in the context of food and eating [90]. However, this was a small-sized qualitative study based on the themes that participants spontaneously mentioned during a semi-structured interview. As we have outlined above, all of the new symptoms can conceivably be applied to eating. Thus, future studies using standardized measures such as a revised YFAS are necessary for appropriately evaluating the relevance of the new SUD criteria for food addiction.

Even if it turns out that the new symptoms, except craving, do not occur in the context of food and eating, it may still be questioned if this would disprove the existence of food addiction. As can be seen in Table 1, the diagnostic criteria as outlined in the DSM-5 do not apply to each substance to the same extent. Specifically, there are SUDs that do not cover the full range of symptoms (caffeine, hallucinogens, inhalants) or do not include intoxication (tobacco). In addition to this, the DSM criteria in general have been criticized for being rather inappropriate for tobacco [94]. Also, the DSM is criticized for its lack of focus on underlying mechanisms, which is a central component of the newly proposed RDoC system. Thus, a major test of the food addiction hypothesis will be to not only focus on the signs and symptoms linking addiction and problematic eating behavior, but also to examine the similarities and differences in the underpinnings of these conditions.

To conclude, we think that the DSM-5 criteria may be valuable for food addiction research, even if some of those symptoms may rarely be endorsed by participants exhibiting addiction-like eating. On the other hand, using those criteria for diagnosing food addiction entails the risk to overestimate the occurrence of food addiction. Thus, future investigations need to take great care that the new SUD criteria are properly translated to food and eating and that reasonable diagnostic thresholds are applied when diagnosing food addiction. Finally, we emphasize the need to think more mechanistically in the evaluation of food addiction by examining the contribution of biological, psychological, and behavioral circuits implicated in addiction to problematic eating behaviors.
